# Causal Relationship Between Vitamin D and Anxiety and Depression: A Bidirectional Mendelian Randomization Study

**DOI:** 10.62641/aep.v54i3.2046

**Published:** 2026-06-15

**Authors:** Ting Wang, Ran Chen, Jiacheng Kong, Qiu Zhang, Shouhong Shu

**Affiliations:** ^1^Department of General Practice, The Peoples Hospital of Tongling City, 244000 Tongling, Anhui, China; ^2^Clinical Laboratory Center, The Peoples Hospital of Tongling City, 244000 Tongling, Anhui, China; ^3^Department of Endocrinology, The First Affiliated Hospital of Anhui Medical University, 230032 Hefei, Anhui, China

**Keywords:** 25-hydroxyvitamin D, Mendelian randomization analysis, depression, anxiety, genetic polymorphism, vitamin D deficiency

## Abstract

**Objective:**

This study aimed to investigate the causal relationship between serum 25-hydroxyvitamin D (25(OH)D) levels and the risk of depression and anxiety, using Mendelian Randomization (MR) analysis from a genetic variation perspective.

**Methods:**

Genome-wide association study (GWAS) summary statistics for 25(OH)D levels, depression and anxiety were retrieved from the IEU Open GWAS Project, specifically from datasets “ebi-a-GCST90000617'', “ebi-a-GCST90013878'', and “ukb-a-82''. A two-sample MR analysis was conducted with 25(OH)D levels as the exposure and depression and anxiety as outcome variables. Cochran's Q test was used to assess heterogeneity among the instrumental variables, and horizontal pleiotropy was tested using the MR-Egger intercept method. Sensitivity analyses were conducted via the leave-one-out approach.

**Results:**

The MR analysis, utilizing 32 single nucleotide polymorphisms (SNPs) as instrumental variables, revealed a significant causal association between 25(OH)D levels and depression, with all MR methods (excluding Simple Mode) yielding *p*-values < 0.05. The odds ratios (ORs) and 95% confidence intervals (CIs) for the significant methods were: OR = 0.82 [95% CI: 0.68–0.98], OR = 0.85 [95% CI: 0.77–0.95], OR = 0.80 [95% CI: 0.68–0.93] and OR = 0.90 [95% CI: 0.82–0.98]. However, no significant causal relationship was found between 25(OH)D levels and anxiety. In the reverse direction, genetically predicted depression showed a potential causal association with lower 25(OH)D levels (inverse variance weighting (IVW) OR = 0.98, 95% CI: 0.96–1.00, *p* = 0.02), while no such association was observed for anxiety.

**Conclusions:**

This study suggests that higher serum 25(OH)D levels may be associated with a lower risk of depression, highlighting the potential of serum 25(OH)D as an early biomarker for depression prevention and clinical management. However, no causal association was found between 25(OH)D levels and anxiety, warranting further investigation. This study provides a potential vitamin D-related intervention direction for the prevention and clinical management of depression.

## Introduction

The rapid pace of modern life, coupled with increasing work-related stress, has subjected individuals to increasingly complex psychological challenges in daily life. At the same time, the widespread use of social media has emerged as a double-edged sword—while it offers a convenient platform for communication, it also introduces unprecedented sources of psychological pressure [1, 2, 3]. Globally, the prevalence of anxiety and depression has risen markedly in recent years. A systematic review published in The Lancet reported that between January 2020 and January 2021, the global prevalence of depression increased from 2470.5 per 100,000 to 3152.9 per 100,000, equating to approximately 53 million new cases—an increase of 27.6%. The prevalence of anxiety disorders similarly rose from 3824.9 to 4802.4 per 100,000, representing an additional 76 million cases and a 25.6% increase [4]. These statistics underscore the widespread and pressing nature of mental health concerns worldwide.

Vitamin D, a fat-soluble vitamin, has gained increasing attention in recent years for its potential role in modulating neurotransmitter balance, immune function, and emotional well-being [5, 6]. A growing body of evidence suggests that adequate vitamin D intake may help prevent the onset of various mental health disorders [7, 8]. For instance, a study by Hemamy *et al*. [9] demonstrated that children receiving vitamin D and magnesium supplementation showed significant improvements across multiple domains, including emotional problems (*p* = 0.001), behavioral issues (*p* = 0.002), peer problems (*p* = 0.001), prosocial behavior (*p* = 0.007), total difficulties (*p* = 0.001), externalizing scores (*p* = 0.001), and internalizing scores (*p* = 0.001). Similarly, research by Głąbska *et al*. [10] on patients with multiple sclerosis indicated that vitamin D supplementation had beneficial effects on psychological outcomes, including quality of life, depression, and fatigue. In another study, Maddock *et al*. [11] reported an inverse association between serum 25-hydroxyvitamin D levels and the risk of depression and panic disorders, even after adjusting for behavioral factors related to vitamin D (OR = 0.57, 95% CI: 0.40–0.81; OR = 0.33, 95% CI: 0.40–0.81).

Genetic and biological factors serve as fundamental underpinnings of mood disorders and should not be overlooked. Evidence from family studies has shown significant clustering of anxiety and depression, highlighting the pivotal role of hereditary influences in their etiology [12, 13]. Specific gene variants, particularly those involved in neurotransmitter synthesis, transport, and receptor function, are recognized as key factors contributing to individual susceptibility to mood disorders. These genetic polymorphisms may disrupt the balance of neurotransmitters in the brain, thereby triggering or exacerbating symptoms of anxiety and depression. Single nucleotide polymorphisms (SNPs), one of the most common types of genetic variation, have been repeatedly implicated in the pathogenesis of mental illnesses. For example, Liu *et al*. [14] identified several SNPs in the TPH2 gene-such as rs4570625, rs17110747, rs120074175, and rs4290270-as significantly associated with depression, with rs11178997 (A/A genotype) emerging as a potential risk factor among Chinese individuals. In a separate study, Liu *et al*. [15] found that the CRH gene’s rs242939 allele (*p* = 0.0008) and genotype (*p* = 0.0002), along with the G-G-T haplotype defined by rs1876828, rs242939, and rs242941, were significantly more prevalent in individuals with major depressive disorder compared to healthy controls. Moreover, research by Keszler *et al*. [16] showed that the T allele of rs1042577, located in the 3^′^ untranslated region of the galanin gene, was associated with higher anxiety levels (HADS score: 7.05 ± 4.0 vs. 6.15 ± 0.15; *p* = 0.000407).

Although existing observational studies suggest an association between vitamin D and mental health, these findings are often confounded by factors such as lifestyle, comorbidities, and environmental exposures [17], as well as reverse causality, making it difficult to establish a causal relationship. Furthermore, many studies are limited by restricted sample sources, limited statistical power [18], and a lack of systematic control for potential genetic backgrounds.

In recent years, Mendelian randomization (MR) analysis has emerged as a powerful causal inference method widely applied in biomedical research. By using genetic variants strongly associated with an exposure (e.g., vitamin D levels) as instrumental variables, MR can effectively minimize confounding biases inherent in conventional observational studies and help infer causal direction [19, 20]. Therefore, applying MR to investigate the potential causal relationship between serum 25-hydroxyvitamin D and common psychological conditions such as anxiety and depression holds significant theoretical value and public health importance. Furthermore, to interrogate the possibility of reverse causation—where mental health status might influence vitamin D levels—we employed a bidirectional MR design.

Taken together, the present study is grounded in the aforementioned research background and applies Mendelian randomization analysis to explore the association between serum 25-hydroxyvitamin D levels and the risk of developing common psychological conditions such as anxiety and depression. The findings aim to offer new insights and robust evidence to support the prevention, early intervention, and clinical management of mental health disorders.

## Materials and Methods

### Study Design

**Fig. 1.**
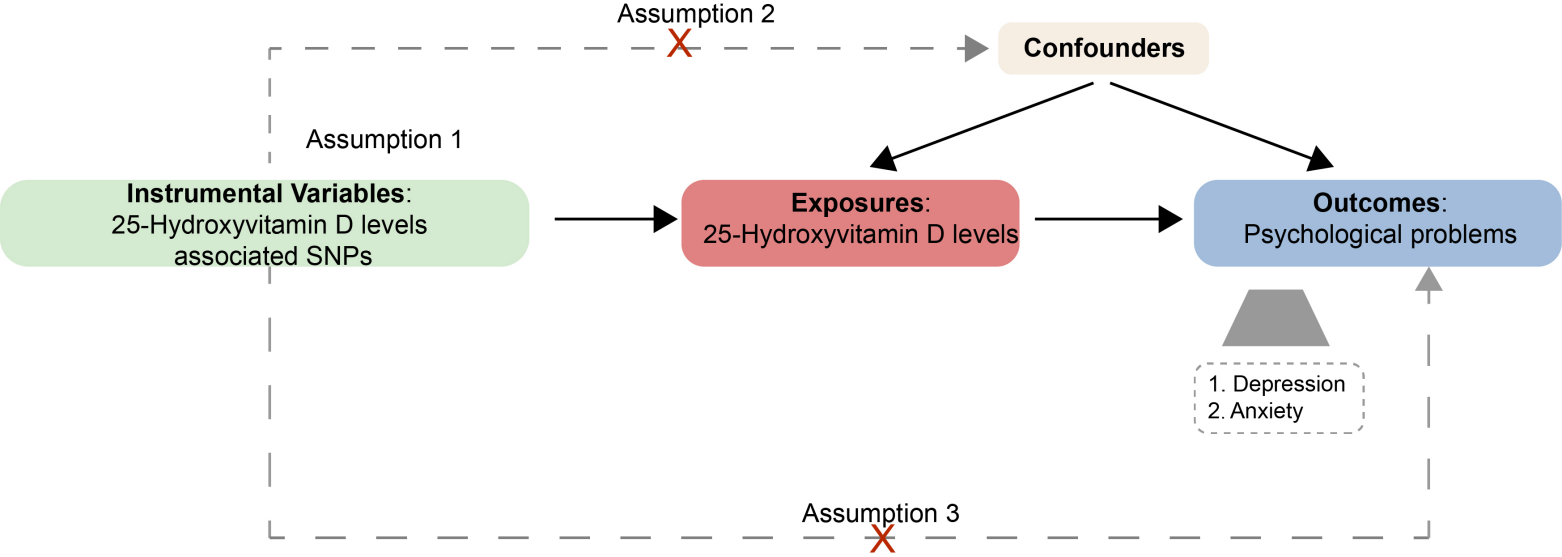
**Conceptual framework of MR analysis of vitamin D, depression and anxiety**. Note: “×” indicates this pathway is excluded.

This Mendelian randomization study aims to investigate the causal relationship between serum 25-hydroxy vitamin D (25(OH)D) levels and the risk of depression and anxiety. The analysis is grounded in three core assumptions: (1) the SNPs selected as instrumental variables are strongly associated with serum 25-hydroxyvitamin D levels and reach the threshold for genome-wide significance; (2) these instrumental variables are independent of potential confounding factors; (3) the instrumental variables influence the risk of psychological disorders exclusively through their effect on serum 25-hydroxyvitamin D levels, without exerting effects via alternative biological pathways (Fig. 1).

### Data Sources

Data on psychological disorders were obtained from the IEU Open GWAS Project (https://gwas.mrcieu.ac.uk/) through a systematic search using the keywords “depression” and “anxiety”. Following screening and selection, the genome-wide association study (GWAS) dataset with ID ebi-a-GCST90013878 was selected for depression, encompassing information on 11,039,205 SNPs from a cohort of 407,746 individuals of European ancestry. For anxiety, the GWAS dataset with ID ukb-a-82 was included, comprising 10,894,596 SNPs derived from 337,159 individuals of European descent.

Data on serum 25-hydroxyvitamin D [25(OH)D] levels were retrieved using the keywords “25(OH)D”, “VD”, “25-Hydroxyvitamin D”, and “1,25-(OH)_2D_3”. The selected dataset, with GWAS ID ebi-a-GCST90000617, contains genetic data on 8,401,108 SNPs from 417,580 individuals of European ancestry. Detailed characteristics of the GWAS datasets are summarized in Table 1 below.

**Table 1.** **Characteristics of the GWAS datasets used in the Mendelian randomization analysis**.

GWAS ID	Trait	Genome build	Population	nSNP	Sample size	ncontrol	ncase	pmid
ebi-a-GCST90013878	Depression (Firth correction)	HG19/GRCh37	European	11,039,205	407,746	NA	NA	34017140
ukb-a-82	Non-cancer illness code self-reported: anxiety/panic attacks	HG19/GRCh37	European	10,894,596	337,159	332,548	4611	NA
ebi-a-GCST90000617	Serum 25-Hydroxyvitamin D levels	HG19/GRCh37	European	8,401,108	417,580	NA	NA	32242144

Note: GWAS, genome-wide association study; SNP, single nucleotide polymorphism; nSNP, number of SNPs; ncontrol, number of controls; ncase, number of cases; pmid, PubMed identifier; NA, not applicable.

### Selection of Relevant SNPs

SNPs associated with the phenotypes of interest were identified based on genome-wide significance thresholds. For the exposure trait (serum 25-hydroxyvitamin D levels), instrumental variables (SNPs) were selected based on a stringent genome-wide significance threshold of *p* < 1 × 10⁻^16^ [21, 22, 23, 24]. This threshold was chosen to ensure that only SNPs with extremely strong associations with vitamin D levels were included, thereby maximizing the strength of the instrumental variables and minimizing the risk of weak instrument bias. To maintain the independence of selected SNPs — commonly defined as linkage disequilibrium (LD) coefficient r^2^ < 0.1 — we applied the clumping procedure using PLINK software (version 1.9.0; PLINK development team, Boston, MA, USA) with a stringent threshold (r^2^ < 0.001, window size = 10,000 kb). In cases where multiple SNPs were in LD, only the SNP with the smallest *p*-value was retained to minimize potential LD-related bias [25, 26].

To evaluate the strength of the selected instrumental variables, the proportion of variance explained (R^2^) and the F-statistic were calculated for each SNP. The R^2^ was computed using the following formula: R^2^ = 2 × MAF × (1 – MAF) × β^2^; where MAF denotes the minor allele frequency and β represents the effect size of the corresponding SNP [27].

**Fig. 2.**
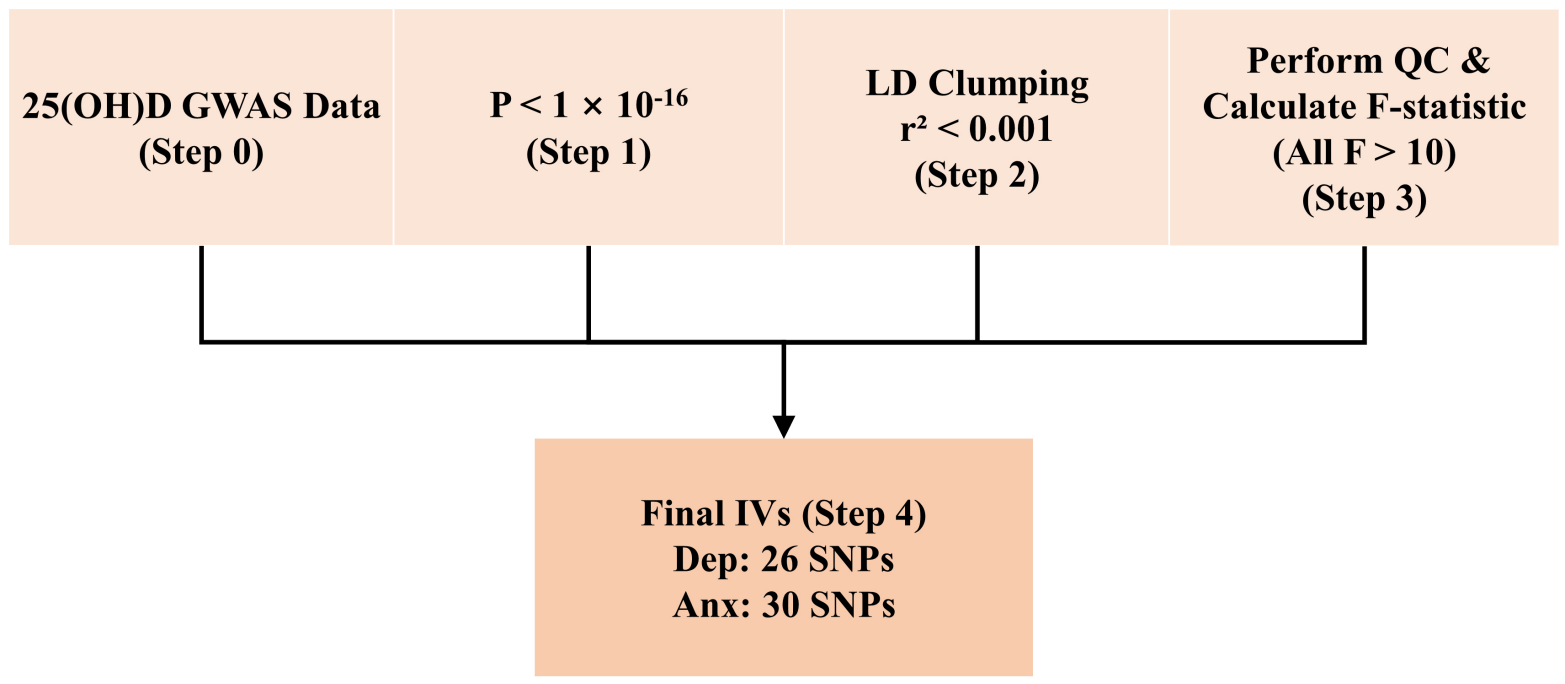
**Simplified workflow for selecting instrumental variables**. Note: GWAS, genome-wide association study; SNP, single nucleotide polymorphism; LD, linkage disequilibrium; IV, instrumental variable; QC, quality control.

In MR analyses, weak instrument bias can arise when SNPs explain only a small fraction of the variance in the exposure, thereby compromising the reliability of causal inference. The F-statistic, which quantifies the strength of association between instrumental variables and the exposure, serves as a critical metric for instrument validity. An R^2^ value approaching zero suggests a weak association, increasing the risk of bias. Generally, an F-statistic below 10 indicates potential weak instrument bias and may undermine the robustness of MR findings. The F-statistic was calculated using the following equation: F = [R^2^ × (N – 2)] / (1 – R^2^); where R^2^ is the coefficient of determination between the exposure (X) and the instrumental variable (Z), reflecting the proportion of exposure variance explained by the instrument; N is the sample size, and N – 2 accounts for degrees of freedom in the calculation [28]. The stepwise process for selecting instrumental variables is summarized in Fig. 2.

### MR Analysis

MR analyses were performed using R software (version 4.3.2; R Foundation for Statistical Computing, Vienna, Austria) with the “TwoSampleMR” package (version 0.6.22; MRC Biostatistics Unit, Cambridge, United Kingdom) and the “MRPRESSO” package (version 1.0; developers affiliated with the MRC Biostatistics Unit, Cambridge, United Kingdom). To investigate the causal relationship between 25-hydroxyvitamin D levels and the occurrence of psychological disorders, we applied a range of analytical methods, including inverse variance weighting (IVW), MR-Egger regression, the weighted median method, simple mode, and the MR-PRESSO algorithm. Cochran’s Q statistic was utilized to assess the heterogeneity among the selected instrumental variables. The MR-Egger approach was further used to test for horizontal pleiotropy. Additionally, sensitivity analysis was conducted using the leave-one-out method to evaluate the robustness of the causal estimates by sequentially removing each SNP. To assess potential reverse causality, bidirectional MR analyses were also conducted with depression and anxiety as exposures and serum 25-hydroxyvitamin D levels as the outcome. For these reverse analyses, instrumental variables for depression and anxiety were selected at a genome-wide significance threshold of *p* < 1 × 10^-5^, followed by the same LD clumping procedure (r^2^ < 0.001, window size = 10,000 kb). The strength of these instruments was evaluated using the F-statistic, with all retained SNPs having F >10. The differential genome-wide significance thresholds applied for selecting instrumental variables in the forward (*p* < 1 × 10^-16^) and reverse (*p* < 1 × 10^-5^) MR analyses were informed by the distinct genetic architectures of the traits. For serum 25(OH)D, a stringent threshold was used to ensure very strong instruments and minimize weak instrument bias, given the typically modest effect sizes of vitamin D-associated SNPs. For depression and anxiety, a slightly more lenient threshold—commonly employed in MR studies of polygenic psychiatric traits—was adopted to retain a sufficient number of strong instruments (all with F-statistic >10) for adequately powered analyses.

## Results

### 25-Hydroxyvitamin D GWAS Data and Instrumental Variable Construction

For the GWAS data on 25-hydroxyvitamin D levels (“ebi-a-GCST90000617”), SNPs were initially selected based on a genome-wide statistical significance threshold (*p* < 1 × 10^-16^) and using the PLINK clumping method with a threshold of r^2^ < 0.001 and a window size of 10,000 kb. A total of 32 relevant SNPs were identified. The R^2^ values and F-statistics for each SNP were subsequently calculated. All SNPs exhibited F-statistics >10, meeting the pre‑defined threshold for strong instrumental variables; therefore, no SNP was excluded due to weak instrument bias (Table  2).

**Table 2.** **SNPs of 25 hydroxyvitamin D genetic tool variables**.

SNPs	EA	OA	β	SE	*p*
rs10859995	C	T	–4.02 × 10^–⁢2^	2.00 × 10^–⁢3^	1.60 × 10^–⁢90^
rs11076175	G	A	2.27 × 10^–⁢2^	2.60 × 10^–⁢3^	1.27 × 10^–⁢18^
rs113209890	T	C	–4.98 × 10^–⁢2^	3.20 × 10^–⁢3^	1.37 × 10^–⁢53^
rs12056768	G	T	–2.16 × 10^–⁢2^	2.00 × 10^–⁢3^	2.82 × 10^–⁢27^
rs12123821	T	C	7.71 × 10^–⁢2^	4.60 × 10^–⁢3^	9.58 × 10^–⁢63^
rs1260326	C	T	2.06 × 10^–⁢2^	2.00 × 10^–⁢3^	1.17 × 10^–⁢24^
rs140589749	T	G	–2.38 × 10^–⁢2^	2.60 × 10^–⁢3^	4.31 × 10^–⁢20^
rs141509989	T	C	–7.55 × 10^–⁢2^	7.60 × 10^–⁢3^	1.84 × 10^–⁢23^
rs142158911	A	G	2.72 × 10^–⁢2^	3.10 × 10^–⁢3^	2.04 × 10^–⁢18^
rs146128209	G	A	–4.55 × 10^–⁢2^	3.80 × 10^–⁢3^	2.44 × 10^–⁢32^
rs1532085	G	A	2.44 × 10^–⁢2^	2.00 × 10^–⁢3^	1.09 × 10^–⁢33^
rs1792287	G	A	2.17 × 10^–⁢2^	2.20 × 10^–⁢3^	3.24 × 10^–⁢22^
rs1800588	T	C	–3.08 × 10^–⁢2^	2.40 × 10^–⁢3^	4.27 × 10^–⁢38^
rs182244780	A	G	–3.27 × 10^–⁢1^	8.70 × 10^–⁢3^	1.00 × 10^–⁢200^
rs2012736	A	C	–4.55 × 10^–⁢2^	3.60 × 10^–⁢3^	2.58 × 10^–⁢36^
rs212100	C	T	–6.03 × 10^–⁢2^	2.70 × 10^–⁢3^	4.44 × 10^–⁢114^
rs2131925	T	G	–2.11 × 10^–⁢2^	2.10 × 10^–⁢3^	9.50 × 10^–⁢25^
rs2352974	T	C	1.68 × 10^–⁢2^	2.00 × 10^–⁢3^	2.22 × 10^–⁢17^
rs2585442	G	C	3.42 × 10^–⁢2^	2.30 × 10^–⁢3^	3.14 × 10^–⁢48^
rs28437159	T	C	5.57 × 10^–⁢2^	2.70 × 10^–⁢3^	1.87 × 10^–⁢92^
rs35408430	T	C	–2.08 × 10^–⁢2^	2.10 × 10^–⁢3^	1.20 × 10^–⁢23^
rs35846253	T	C	–6.15 × 10^–⁢2^	2.50 × 10^–⁢3^	7.71 × 10^–⁢130^
rs4536175	T	C	–5.94 × 10^–⁢2^	2.10 × 10^–⁢3^	5.94 × 10^–⁢181^
rs6123359	G	A	3.18 × 10^–⁢2^	3.30 × 10^–⁢3^	2.04 × 10^–⁢22^
rs61815559	T	A	8.31 × 10^–⁢2^	5.70 × 10^–⁢3^	6.56 × 10^–⁢49^
rs6782190	A	G	–1.89 × 10^–⁢2^	2.10 × 10^–⁢3^	3.72 × 10^–⁢20^
rs6837680	T	A	7.41 × 10^–⁢2^	2.10 × 10^–⁢3^	1.00 × 10^–⁢200^
rs736894	T	C	–9.78 × 10^–⁢2^	2.50 × 10^–⁢3^	1.00 × 10^–⁢200^
rs7528419	G	A	1.99 × 10^–⁢2^	2.40 × 10^–⁢3^	3.35 × 10^–⁢17^
rs8018720	C	G	–3.00 × 10^–⁢2^	2.60 × 10^–⁢3^	2.63 × 10^–⁢31^
rs8107974	T	A	3.58 × 10^–⁢2^	3.70 × 10^–⁢3^	3.69 × 10^–⁢22^
rs964184	C	G	4.14 × 10^–⁢2^	2.90 × 10^–⁢3^	4.39 × 10^–⁢46^

Note: SNP, single nucleotide polymorphism; EA, effect allele; OA, other allele; β, beta coefficient; SE, standard error; *p*, *p*-value.

### MR Analysis of 25-Hydroxyvitamin D Levels and Depression

**Fig. 3.**
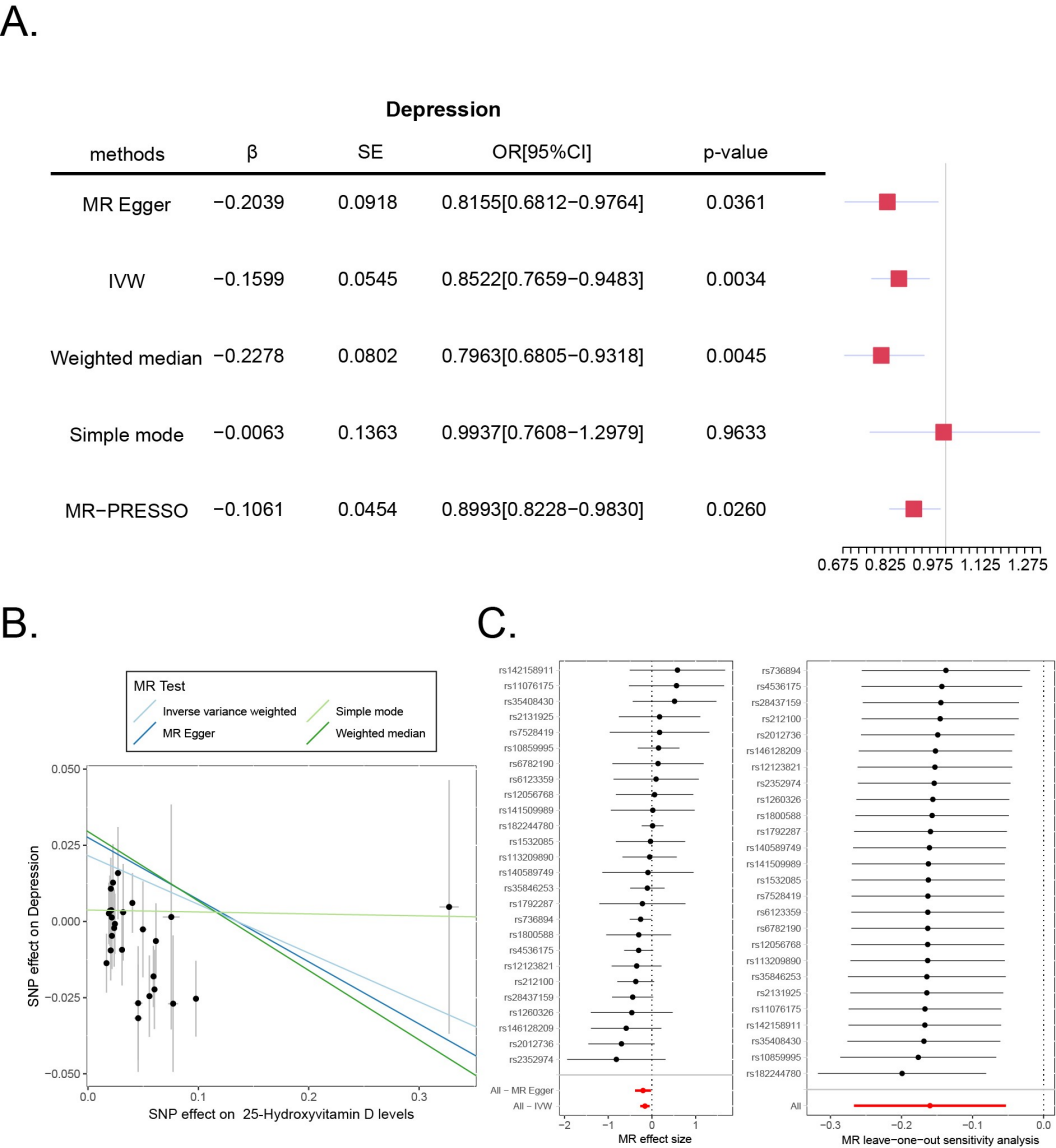
**MR analysis results of 25 hydroxyvitamin D level and depression**. (A) Sensitivity analysis of causal effect estimates (OR, 95% CI) from five MR methods. (B) Scatter plot of SNP effects on 25-hydroxyvitamin D vs. depression, with slopes from different MR methods. (C) Robustness checks: individual SNP forest plot (left) and leave-one-out analysis (right). Note: OR, Odds Ratio; CI, Confidence Interval; se, Standard Error; *p*-value > 0.05 indicates no statistical significance.

In the GWAS data associated with depression (“ebi-a-GCST90013878”), a total of 32 relevant SNPs were identified. After excluding 6 low-quality SNPs (rs2585442, rs61815559, rs6837680, rs8018720, rs8107974, rs964184) based on allele frequencies and linkage disequilibrium effects, 26 SNPs were retained as instrumental variables for subsequent analysis (see **Supplementary Table 1** in the **Supplementary Materials**). The results of the MR analysis revealed that, with the exception of the Simple mode, all tests had *p*-values < 0.05, with odds ratios (OR) [95% CI] of 0.82 [0.68–0.98], 0.85 [0.77–0.95], 0.80 [0.68–0.93], and 0.90 [0.82–0.98], respectively. These findings indicate that 25-hydroxyvitamin D levels are a significant risk factor for the development of depression. Cochran’s Q-test for the MR-Egger and IVW algorithms yielded *p*-values of 0.72 and 0.75, respectively, indicating no heterogeneity between the two methods. Additionally, the horizontal pleiotropy test for the MR-Egger method returned a *p*-value of 0.56, suggesting no evidence of pleiotropy (Table 3 and Fig. 3). And the result of Leave-One-Out analysis showed that instrumental variables have stability (Fig. 3C).

**Table 3.** **MR analysis of 25 hydroxyvitamin D level and depression**.

MR info					Cochran’s Q			Horizontal pleiotropy		
Method	β	SE	OR [95% CI]	*p*val	Q	Q_df	Q_*p*val	Intercept	SE	*p*
MR-Egger	–2.04 × 10^–⁢1^	9.18 × 10^–⁢2^	0.82 [0.68–0.98]	0.04	19.61	24	0.72	<0.01	<0.01	0.56
IVW	–1.60 × 10^–⁢1^	5.45 × 10^–⁢2^	0.85 [0.77–0.95]	<0.01	19.96	25	0.75			
Weighted median	–2.28 × 10^–⁢1^	8.02 × 10^–⁢2^	0.80 [0.68–0.93]	<0.01	-	-	-			
Simple mode	–6.30 × 10^–⁢3^	1.36 × 10^–⁢1^	0.99 [0.76–1.30]	0.96	-	-	-			
MR-PRESSO	–1.06 × 10^–⁢1^	4.54 × 10^–⁢2^	0.90 [0.82–0.98]	0.03	-	-	-			

Note: MR, Mendelian randomization; SE, standard error; OR, odds ratio; CI, confidence interval; IVW, inverse variance weighted; Q, Cochran’s Q statistic for heterogeneity; Q_df, degrees of freedom for Q; Q_*p*val, *p*-value for the Q statistic.

### MR Analysis of 25-Hydroxyvitamin D Levels and Anxiety

**Fig. 4.**
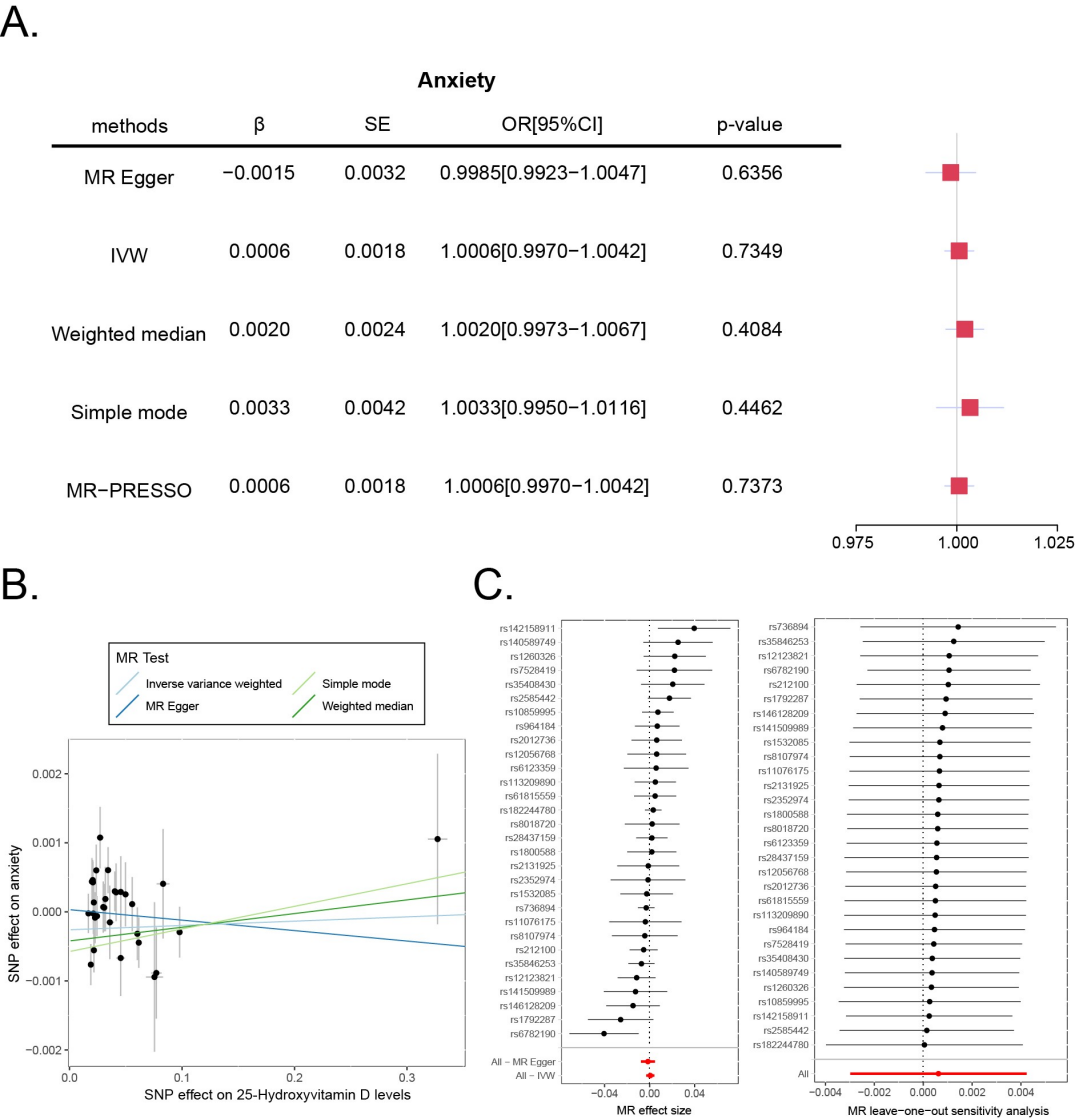
**MR analysis results of 25 hydroxyvitamin D level and the occurrence of anxiety disorder**. (A) Sensitivity analysis of causal effect estimates (OR, 95% CI) from five MR methods. (B) Scatter plot of SNP effects on 25-hydroxyvitamin D vs. anxiety, with slopes from different MR methods. (C) Robustness checks: individual SNP forest plot (left) and leave-one-out analysis (right). Note: OR <1 suggests higher 25-hydroxyvitamin D levels are associated with a reduced risk of anxiety; *p*-value < 0.05 indicates statistical significance.

In the GWAS data related to anxiety (“ukb-a-82”), a total of 30 relevant SNPs were identified. After excluding no low-quality SNPs based on allele frequencies and linkage disequilibrium effects, all 30 SNPs were retained as instrumental variables for subsequent analysis (see **Supplementary Table 2** in the **Supplementary Materials**). A two-sample MR analysis was conducted to investigate the association between 25-hydroxyvitamin D levels and the occurrence of anxiety. The results revealed that the *p*-values for all five MR algorithms were greater than 0.05, indicating no statistical significance. Cochran’s Q-test for the MR-Egger and IVW algorithms yielded *p*-values of 0.11 and 0.12, respectively, suggesting no heterogeneity between the two methods. Additionally, the horizontal pleiotropy test for the MR-Egger method produced a *p*-value of 0.41, indicating no evidence of pleiotropy. (Table 4 and Fig. 4). And the result of Leave-One-Out analysis showed that instrumental variables have stability (Fig. 4C).

**Table 4.** **MR analysis of 25 hydroxyvitamin D level and anxiety**.

MR info					Cochran’s Q			Horizontal pleiotropy		
Method	β	SE	OR [95% CI]	*p*val	Q	Q_df	Q_*p*val	Intercept	SE	*p*
MR-Egger	–1.50 × 10^–⁢3^	3.20 × 10^–⁢3^	1.00 [0.99–1.00]	0.64	37.24	28	0.11	1.00 × 10^–⁢4^	1.00 × 10^–⁢4^	0.41
IVW	6.00 × 10^–⁢4^	1.80 × 10^–⁢3^	1.00 [1.00 –1.00]	0.73	38.17	29	0.12	-	-	-
Weighted median	2.00 × 10^–⁢3^	2.40 × 10^–⁢3^	1.00 [1.00–1.01]	0.41	-	-	-	-	-	-
Simple mode	3.30 × 10^–⁢3^	4.20 × 10^–⁢3^	1.00 [1.00–1.01]	0.45	-	-	-	-	-	-
MR-PRESSO	6.00 × 10^–⁢4^	1.80 × 10^–⁢3^	1.00 [1.00–1.00]	0.74	-	-	-	-	-	-

Note: MR, Mendelian randomization; SE, standard error; OR, odds ratio; CI, confidence interval; IVW, inverse variance weighted; Q, Cochran’s Q statistic for heterogeneity; Q_df, degrees of freedom for Q; Q_*p*val, *p*-value for the Q statistic.

### Reverse MR Analysis: Effect of Depression and Anxiety on 25-Hydroxyvitamin D Levels

To examine the possibility of reverse causality, we performed MR analyses with depression and anxiety as exposures and serum 25(OH)D levels as the outcome.

For depression, 52 independent SNPs were selected as instruments. After harmonization with the 25(OH)D outcome dataset, 44 SNPs were retained for analysis. The IVW method indicated a potential causal effect of depression on lower 25(OH)D levels (OR = 0.98, 95% CI: 0.96–1.00, *p* = 0.02). The MR-PRESSO method yielded a similar estimate (OR = 0.98, 95% CI: 0.96–1.00, *p* = 0.03). However, the MR-Egger intercept test did not suggest significant horizontal pleiotropy (*p* = 0.66), and the weighted median estimate was not statistically significant (OR = 0.99, 95% CI: 0.97–1.01, *p* = 0.17). Notable heterogeneity was observed among the instruments (Cochran’s Q *p* < 0.001).

For anxiety, 30 SNPs were selected, with 25 retained after harmonization. None of the MR methods provided evidence for a causal effect of anxiety on 25(OH)D levels (IVW OR = 1.23, 95% CI: 0.68–2.22, *p* = 0.49). No significant heterogeneity or horizontal pleiotropy was detected for the anxiety analysis (Table 5, see **Supplementary Figs. 1,2** in the **Supplementary Materials**).

**Table 5.** **MR analysis of depression or anxiety on serum 25-hydroxyvitamin D levels**.

MR info							Cochran’s Q			Horizontal pleiotropy		
Exposures	SNP (n)	Method	β	SE	OR [95% CI]	*p*val	Q	Q_df	Q_*p*val	Intercept	SE	*p*
Depression	44	MR-Egger	–2.97 × 10^–⁢2^	2.39 × 10^–⁢2^	0.97 [0.93–1.02]	0.22	78.25	42	0.0006	7.00 × 10^–⁢4^	1.50 × 10^–⁢3^	0.66
		IVW	–1.99 × 10^–⁢2^	8.80 × 10^–⁢3^	0.98 [0.96–1.00]	0.02	78.62	43	0.0007	-	-	-
		Weighted median	–1.35 × 10^–⁢2^	9.80 × 10^–⁢3^	0.99 [0.97–1.01]	0.17	-	-	-	-	-	-
		Simple mode	–4.10 × 10^–⁢3^	2.06 × 10^–⁢2^	1.00 [0.96–1.04]	0.84	-	-	-	-	-	-
		MR-PRESSO	–1.99 × 10^–⁢2^	8.80 × 10^–⁢3^	0.98 [0.96–1.00]	0.03	-	-	-	-	-	-
Anxiety	25	MR-Egger	1.70 × 10^–⁢2^	7.12 × 10^–⁢1^	1.02 [0.25–4.10]	0.98	23.03	23	0.46	4.00 × 10^–⁢4^	1.40 × 10^–⁢3^	0.77
		IVW	2.06 × 10^–⁢1^	3.01 × 10^–⁢1^	1.23 [0.68–2.22]	0.49	23.11	24	0.51	-	-	-
		Weighted median	–1.08 × 10^–⁢1^	4.29 × 10^–⁢1^	0.90 [0.39–2.08]	0.80	-	-	-	-	-	-
		Simple mode	–8.43 × 10^–⁢1^	9.19 × 10^–⁢1^	0.43 [0.07–2.61]	0.37	-	-	-	-	-	-
		MR-PRESSO	2.06 × 10^–⁢1^	2.95 × 10^–⁢1^	1.23 [0.69–2.19]	0.49	-	-	-	-	-	-

Note: MR, Mendelian randomization; SE, standard error; OR, odds ratio; CI, confidence interval; IVW, inverse variance weighted; Q, Cochran’s Q statistic for heterogeneity; Q_df, degrees of freedom for Q; Q_*p*val, *p*-value for the Q statistic.

## Discussion

In recent years, with the growing public attention to mental health, the potential association between vitamin D and psychological disorders such as depression and anxiety has become a focus of clinical research [29, 30]. Vitamin D primarily exists in two forms: vitamin D_3_ (cholecalciferol) and vitamin D_2_ (ergocalciferol). In the skin, 7-dehydrocholesterol is converted into the precursor of vitamin D_3_ under ultraviolet B (UVB, 290–320 nm) radiation, which is then further metabolized in the liver and kidneys to its active form, 1,25-(OH)_2_ -D_3_[31]. In addition to endogenous synthesis, vitamin D can also be obtained through dietary sources (such as fish, egg yolks, and dairy products) or supplements.

Numerous studies have demonstrated a close relationship between vitamin D status, commonly reflected by serum 25(OH)D levels, and various psychological disorders, including depression, anxiety, and mood disturbances [32]. Zhao *et al*. [33] using a convenience sampling method, conducted a cross-sectional study involving 1323 students from four middle schools in Shenzhen to explore the association between vitamin D deficiency and anxiety, depression, and comorbid symptoms. Their findings indicated that vitamin D deficiency, typically defined by low serum 25(OH)D concentrations, was a risk factor for anxiety (OR = 1.59, 95% CI: 1.02–2.46), depression (OR = 1.94, 95% CI: 1.22–3.09), and anxiety-depression comorbidity (OR = 1.75, 95% CI: 1.06–2.90).

An umbrella meta-analysis by Musazadeh *et al*. [34] revealed that participants receiving vitamin D supplementation exhibited significantly reduced depressive symptoms (combined standardized mean difference = –0.40). Furthermore, individuals with lower serum vitamin D levels were found to have a higher likelihood of depression compared to those with higher vitamin D levels (combined odds ratio: 1.60; 95% CI: 1.08–2.36, *p* < 0.01; I^2^ = 91.3%, *p* < 0.01). Similarly, a study by Xie *et al*. [35] found that vitamin D supplementation was beneficial in reducing the incidence of depression and improving the treatment of depression, with daily doses greater than 2800 IU and durations of at least 8 weeks considered highly effective. However, Borges-Vieira and Cardoso [36] argued that while vitamin D supplementation may aid in alleviating depression, the improvement in symptoms cannot be solely attributed to vitamin D, and that clinical interventions should be more tailored to the patient’s clinical condition and nutritional biomarkers.

Another meta-analysis of 18 prior studies involving 1980 participants showed that vitamin D supplementation significantly alleviated depressive symptoms [37], especially in the early stages of depression. These studies indicate a close relationship between 25-hydroxyvitamin D levels and the onset of depression and anxiety. However, the causal relationship between the two remains to be further clarified by MR analysis.

In the present study, MR analysis was employed to investigate the causal relationship between 25-hydroxyvitamin D levels and the occurrence of depression and anxiety. The results suggest that 25-hydroxyvitamin D levels are causally linked to the onset of depression. Specifically, the MR analysis showed that for all methods, except the Simple mode, the *p*-values were <0.05, and Cochran’s Q-test indicated no heterogeneity between MR-Egger and IVW algorithms (*p*-values of 0.72 and 0.75, respectively). Moreover, the horizontal pleiotropy test for MR-Egger yielded a *p*-value of 0.56, indicating no pleiotropy. These results suggest that higher 25-hydroxyvitamin D levels are associated with a reduced risk of depression.

Our bidirectional MR design allowed us to explore reverse causality. The reverse MR analysis suggested a potential association between genetic liability to depression and lower serum 25(OH)D levels. This finding is consistent with the hypothesis that depression might lead to reduced outdoor activity, altered diet, or other behavioral changes that decrease vitamin D synthesis and intake. However, this result should be interpreted with caution due to the observed heterogeneity among the instrumental variables for depression. In contrast, we found no evidence that genetic predisposition to anxiety influences 25(OH)D levels. These results, taken together with the primary forward analysis, strengthen the inference that the observed association is more likely driven by 25(OH)D status influencing depression risk, rather than the converse.

However, existing MR studies on the causal relationship between 25-hydroxyvitamin D levels and depression report conflicting results. Bassett *et al*. [38] performed both linear and nonlinear MR analyses based on data from the UK Biobank, finding no association between genetically predicted 25(OH)D levels and lifetime depression (OR = 0.97, 95% CI: 0.93–1.01) in linear analysis. However, nonlinear analysis revealed that in the lowest 25% of the population with genetically predicted 25(OH)D levels, there was an association with lifetime depression (OR = 0.75, 95% CI: 0.59–0.94), suggesting that targeted supplementation with 25(OH)D may help reduce the risk of depression. Mulugeta *et al*. [39] used data from 307,618 White British participants in the UK Biobank and other studies, conducting bidirectional MR analysis and found no association between serum 25(OH)D and depression (OR = 0.97, 95% CI: 0.90–1.05), but noted that genetic susceptibility to depression was associated with lower 25(OH)D levels. This study indicated that while depression may contribute to lower 25(OH)D levels, the causal impact of vitamin D status on depression risk remains inconclusive.

Furthermore, Arathimos *et al*. [40] conducted an MR analysis on treatment-resistant depression and atypical depression, finding no genetic evidence supporting a causal relationship between serum 25(OH)D levels and treatment-resistant (NCASE = 1891, OR = 1.01 [95% CI: 0.78–1.31]) or atypical depression (NCASE = 2101, OR = 1.04 [95% CI: 0.80–1.36]). These conflicting results underscore the need for further research to definitively establish the causal relationship between 25-hydroxyvitamin D levels and depression.

As for the MR analysis of anxiety, none of the five MR methods tested yielded statistically significant results (*p*-values > 0.05). Cochran’s Q-test for MR-Egger and IVW algorithms returned *p*-values of 0.11 and 0.12, respectively, suggesting no heterogeneity. The pleiotropy test for MR-Egger showed a *p*-value of 0.41, indicating no pleiotropy. Although no significant causal relationship between 25-hydroxyvitamin D levels and anxiety was observed in this study, this does not imply that vitamin D has no role in anxiety disorders. Previous research by Liu *et al*. [41] found that individuals with generalized anxiety disorder (GAD) had significantly lower serum 25-hydroxyvitamin D levels compared to healthy controls, and that lower vitamin D levels were associated with poorer cognitive function and worse treatment outcomes. The lack of a significant causal relationship between vitamin D levels and anxiety in our study may be due to the complex nature of anxiety disorders, which likely involve a combination of biological, psychological, and social factors. Differences in study definitions, diagnostic criteria, and limitations such as sample size and study design may also influence the findings. Thus, future research with larger sample sizes, more refined classification systems, and stricter diagnostic criteria is needed to explore the potential relationship between vitamin D and anxiety.

This study investigated the causal relationship between serum 25-hydroxyvitamin D levels and the risk of depression and anxiety from a genetic perspective, employing MR analysis. The findings provide statistical support for the potential role of vitamin D supplementation in the prevention and management of psychological disorders. MR analysis, which has benefitted significantly from the development of large-scale biobanks in recent years, offers several advantages over conventional clinical studies with limited sample sizes. These advantages include high-throughput detection of single nucleotide polymorphisms (SNPs), larger and more representative sample populations, and improved statistical power.

Nevertheless, several limitations should be acknowledged. First, the MR analysis is inherently dependent on the availability of large-scale GWAS datasets and specific genetic variants. Due to data constraints, the present study was restricted to individuals of European ancestry, which may limit the generalizability of the findings. Specific gene variants, particularly those involved in neurotransmitter synthesis, transport, and receptor function, are recognized as key factors contributing to individual susceptibility to mood disorders. Second, individual-level genetic data were not available, and GWAS summary statistics stratified by sex and age were lacking. Consequently, this study was unable to explore the potential heterogeneity in the causal associations between vitamin D levels and mental health outcomes across demographic subgroups. Previous research has highlighted that older adults are more likely to have lower vitamin D levels, and that female patients with depression are at elevated risk for cardiovascular events such as myocardial infarction, suggesting that stratified analyses may yield valuable insights. Third, psychiatric disorders such as depression and anxiety are complex conditions involving intricate interactions between genetic and environmental factors. While MR analysis helps to mitigate confounding, it may not fully capture the causal complexity of the relationship between vitamin D status (as indicated by serum 25(OH)D) and mental health. Fourth, our findings are based exclusively on GWAS data from individuals of European ancestry. Future studies using genetic data from diverse ancestral backgrounds (e.g., African, Asian, Hispanic) are needed to assess the generalizability of the observed causal relationship, especially given known population differences in vitamin D metabolism related to skin pigmentation and geographic latitude. Although reverse causation—whereby mental health disorders might influence vitamin D metabolism—is a theoretical concern, our reverse-direction MR analysis did not support a significant causal effect of depression on serum 25(OH)D levels. This finding strengthens the inference that the observed association is more likely driven by vitamin D status influencing depression risk, rather than the converse. Research on the relationship between vitamin D status, particularly as measured by serum 25(OH)D, and mental health is still at a nascent stage. Future studies should aim to include larger, more diverse populations encompassing various regions, ethnic backgrounds, and age groups to enhance the generalizability of findings. Further mechanistic research is also warranted to elucidate the metabolic pathways, biological functions, and molecular mechanisms through which vitamin D influences psychological well-being. This includes exploring its interactions with neurotransmitters, inflammatory responses, and other neurobiological factors. Special attention should be given to the role of vitamin D in regulating neurotransmitter activity, promoting neuroprotection and neuroplasticity, and to the distribution and function of vitamin D receptors in neurons and glial cells, as well as their involvement in neurotransmitter synthesis and release.

Moreover, most existing studies have focused on the short- and medium-term effects of vitamin D on mental health, while its long-term impact remains inadequately explored. Longitudinal follow-up studies are needed to determine whether prolonged vitamin D supplementation can produce sustained improvements in psychological outcomes and to evaluate its long-term efficacy in mental health promotion and disease prevention.

## Conclusion

This study suggests a causal association between serum 25-hydroxyvitamin D levels and depression. Specifically, genetically predicted higher levels were associated with a lower risk of developing the disorder. However, no significant causal association was observed between 25-hydroxyvitamin D levels and the occurrence of anxiety. These findings provide important insights for the clinical understanding of the role and underlying mechanisms of vitamin D, particularly through its circulating form 25(OH)D, in mental health.

## Availability of Data and Materials

The produced and analysed datasets are obtained in the IEU OpenGWAS Project repository [PERSISTENT WEB LINK, https://gwas.mrcieu.ac.uk/]. The accession numbers are: ebi-a-GCST90013878, ukb-a-82 and ebi-a-GCST90000617.

## Supplementary Material

Supplementary material associated with this article can be found, in the online version, at https://doi.org/10.62641/aep.v54i3.2046.
