# Supplementary material for: Causal Relationship Between Vitamin D and Anxiety and Depression: A Bidirectional Mendelian Randomization Study
Source: Actas Esp Psiquiatr. 2026 Jun 15;54(3):878–90. doi: 10.62641/aep.v54i3.2046 (PMC13294854; doi:10.62641/aep.v54i3.2046)
Supplement: Supplementary file 1 [file ActEsp-54-3-878-890-s1.zip › Supplementary_Materials.docx]

**Supplementary Materials Index**

This document contains supplementary tables and figures for the manuscript entitled **“Causal Relationship Between Vitamin D and Anxiety and Depression: A Bidirectional Mendelian Randomization Study”**.

The supplementary materials are organized as follows:

**Supplementary Table 1. Information on the 26 vitamin D–related SNPs used as instrumental variables in the depression analysis.**

***(Corresponds to the section “MR Analysis of 25-Hydroxyvitamin D Levels and Depression” in the main text.)***

**Supplementary Table 2. Information on the 30 vitamin D–related SNPs used as instrumental variables in the anxiety analysis.**

***(Corresponds to the section “MR Analysis of 25-Hydroxyvitamin D Levels and Anxiety” in the main text.)***

**Supplementary Figure 1. Reverse MR analysis of the effect of genetic liability to depression on serum 25-hydroxyvitamin D [25(OH)D] levels.**

***(Corresponds to the “Reverse MR Analysis” section in the main text.)***

**Supplementary Figure 2. Reverse MR analysis of the effect of genetic liability to anxiety on serum 25-hydroxyvitamin D [25(OH)D] levels.**

***(Corresponds to the “Reverse MR Analysis” section in the main text.)***

**Supplementary table 1. The information of 26 vitamin-D relevant SNPs for depression**

| SNP | effect size (β) | standard error (SE) | p-value | R² | F |
| --- | --- | --- | --- | --- | --- |
| rs10859995 | -0.0402 | 0.0020 | 1.60 × 10^-90^ | 0.0312 | 636.4576 |
| rs11076175 | 0.0227 | 0.0026 | 1.27 × 10^-18^ | 0.0136 | 971.7393 |
| rs113209890 | -0.0498 | 0.0032 | 1.37 × 10^-53^ | 0.0238 | 730.9056 |
| rs12056768 | -0.0216 | 0.0020 | 2.82 × 10^-27^ | 0.0167 | 875.4799 |
| rs12123821 | 0.0771 | 0.0046 | 9.58 × 10^-63^ | 0.0259 | 701.0415 |
| rs1260326 | 0.0206 | 0.0020 | 1.17 × 10^-24^ | 0.0159 | 899.7865 |
| rs140589749 | -0.0238 | 0.0026 | 4.31 × 10^-20^ | 0.0142 | 951.5974 |
| rs141509989 | -0.0755 | 0.0076 | 1.84 × 10^-23^ | 0.0154 | 912.0509 |
| rs142158911 | 0.0272 | 0.0031 | 2.04 × 10^-18^ | 0.0135 | 974.7306 |
| rs146128209 | -0.0455 | 0.0038 | 2.44 × 10^-32^ | 0.0183 | 836.2147 |
| rs1532085 | 0.0244 | 0.0020 | 1.09 × 10^-33^ | 0.0187 | 827.0964 |
| rs1792287 | 0.0217 | 0.0022 | 3.24 × 10^-22^ | 0.0150 | 925.7340 |
| rs1800588 | -0.0308 | 0.0024 | 4.27 × 10^-38^ | 0.0200 | 800.3203 |
| rs182244780 | -0.3271 | 0.0087 | 1.00 × 10^-200^ | 0.0467 | 515.9405 |
| rs2012736 | -0.0455 | 0.0036 | 2.58 × 10^-36^ | 0.0195 | 810.6258 |
| rs212100 | -0.0603 | 0.0027 | 4.44 × 10^-114^ | 0.0351 | 598.8505 |
| rs2131925 | -0.0211 | 0.0021 | 9.50 × 10^-25^ | 0.0159 | 898.8749 |
| rs2352974 | 0.0168 | 0.0020 | 2.22 × 10^-17^ | 0.0131 | 990.5365 |
| rs28437159 | 0.0557 | 0.0027 | 1.87 × 10^-92^ | 0.0315 | 632.9269 |
| rs35408430 | -0.0208 | 0.0021 | 1.20 × 10^-23^ | 0.0155 | 910.0668 |
| rs35846253 | -0.0615 | 0.0025 | 7.71 × 10^-130^ | 0.0375 | 578.7860 |
| rs4536175 | -0.0594 | 0.0021 | 5.94 × 10^-181^ | 0.0443 | 530.2761 |
| rs6123359 | 0.0318 | 0.0033 | 2.04 × 10^-22^ | 0.0151 | 923.4616 |
| rs6782190 | -0.0189 | 0.0021 | 3.72 × 10^-20^ | 0.0142 | 950.7754 |
| rs736894 | -0.0978 | 0.0025 | 1.00 × 10^-200^ | 0.0467 | 515.9405 |
| rs7528419 | 0.0199 | 0.0024 | 3.35 × 10^-17^ | 0.0131 | 993.3896 |

Note: SNP = single nucleotide polymorphism; β = beta coefficient; SE = standard error; R² = proportion of variance explained by the SNP in the exposure; F = F-statistic for instrument strength.

**Supplementary table 2. The information of 30 vitamin-D relevant SNPs for anxiety**

| SNP | effect size (β) | standard error (SE) | p-value | R² | F |
| --- | --- | --- | --- | --- | --- |
| rs10859995 | -0.0003 | 0.0003 | 0.3026 | 0.0016 | 2951.2703 |
| rs11076175 | -0.0001 | 0.0004 | 0.8182 | 0.0004 | 6254.1790 |
| rs112072036 | -0.0008 | 0.0007 | 0.2273 | 0.0019 | 2726.8520 |
| rs12056768 | -0.0001 | 0.0003 | 0.6367 | 0.0007 | 4362.3354 |
| rs12123821 | -0.0009 | 0.0007 | 0.1800 | 0.0021 | 2587.2253 |
| rs1260326 | 0.0005 | 0.0003 | 0.1123 | 0.0025 | 2376.9643 |
| rs12803256 | -0.0005 | 0.0003 | 0.1642 | 0.0022 | 2539.9408 |
| rs142158911 | 0.0011 | 0.0004 | 0.0158 | 0.0037 | 1926.8337 |
| rs1532085 | -0.0001 | 0.0003 | 0.8300 | 0.0003 | 6471.1855 |
| rs1660870 | -0.0002 | 0.0003 | 0.4116 | 0.0013 | 3307.6085 |
| rs1800588 | -0.0001 | 0.0003 | 0.8605 | 0.0003 | 7152.2890 |
| rs1993116 | -0.0004 | 0.0003 | 0.1319 | 0.0023 | 2440.4118 |
| rs2012736 | -0.0003 | 0.0005 | 0.5787 | 0.0009 | 4022.6840 |
| rs212100 | 0.0003 | 0.0004 | 0.4054 | 0.0013 | 3285.7353 |
| rs2131925 | 0.0000 | 0.0003 | 0.9329 | 0.0001 | 10,334.3239 |
| rs2352974 | 0.0000 | 0.0003 | 0.9347 | 0.0001 | 10,473.6310 |
| rs2585442 | 0.0006 | 0.0003 | 0.0699 | 0.0028 | 2224.3672 |
| rs35408430 | -0.0004 | 0.0003 | 0.1525 | 0.0022 | 2504.6023 |
| rs4306928 | 0.0005 | 0.0004 | 0.2137 | 0.0019 | 2686.7715 |
| rs4694423 | 0.0001 | 0.0003 | 0.6564 | 0.0007 | 4494.3796 |
| rs6123359 | 0.0002 | 0.0005 | 0.6911 | 0.0006 | 4755.6959 |
| rs61815559 | 0.0004 | 0.0008 | 0.6089 | 0.0008 | 4190.8381 |
| rs6782190 | 0.0008 | 0.0003 | 0.0099 | 0.0040 | 1863.1871 |
| rs71601787 | 0.0003 | 0.0003 | 0.3254 | 0.0015 | 3022.0236 |
| rs72868074 | 0.0007 | 0.0007 | 0.3075 | 0.0016 | 2966.5350 |
| rs7439366 | -0.0003 | 0.0003 | 0.2753 | 0.0017 | 2868.8965 |
| rs7528419 | 0.0004 | 0.0003 | 0.1980 | 0.0020 | 2640.7541 |
| rs8018720 | -0.0001 | 0.0004 | 0.8531 | 0.0003 | 6968.1174 |
| rs8107974 | -0.0002 | 0.0005 | 0.7745 | 0.0004 | 5602.1453 |
| rs964184 | 0.0003 | 0.0004 | 0.4998 | 0.0010 | 3648.6982 |

Note: SNP = single nucleotide polymorphism; β = beta coefficient; SE = standard error; R² = proportion of variance explained by the SNP in the exposure; F = F-statistic for instrument strength.


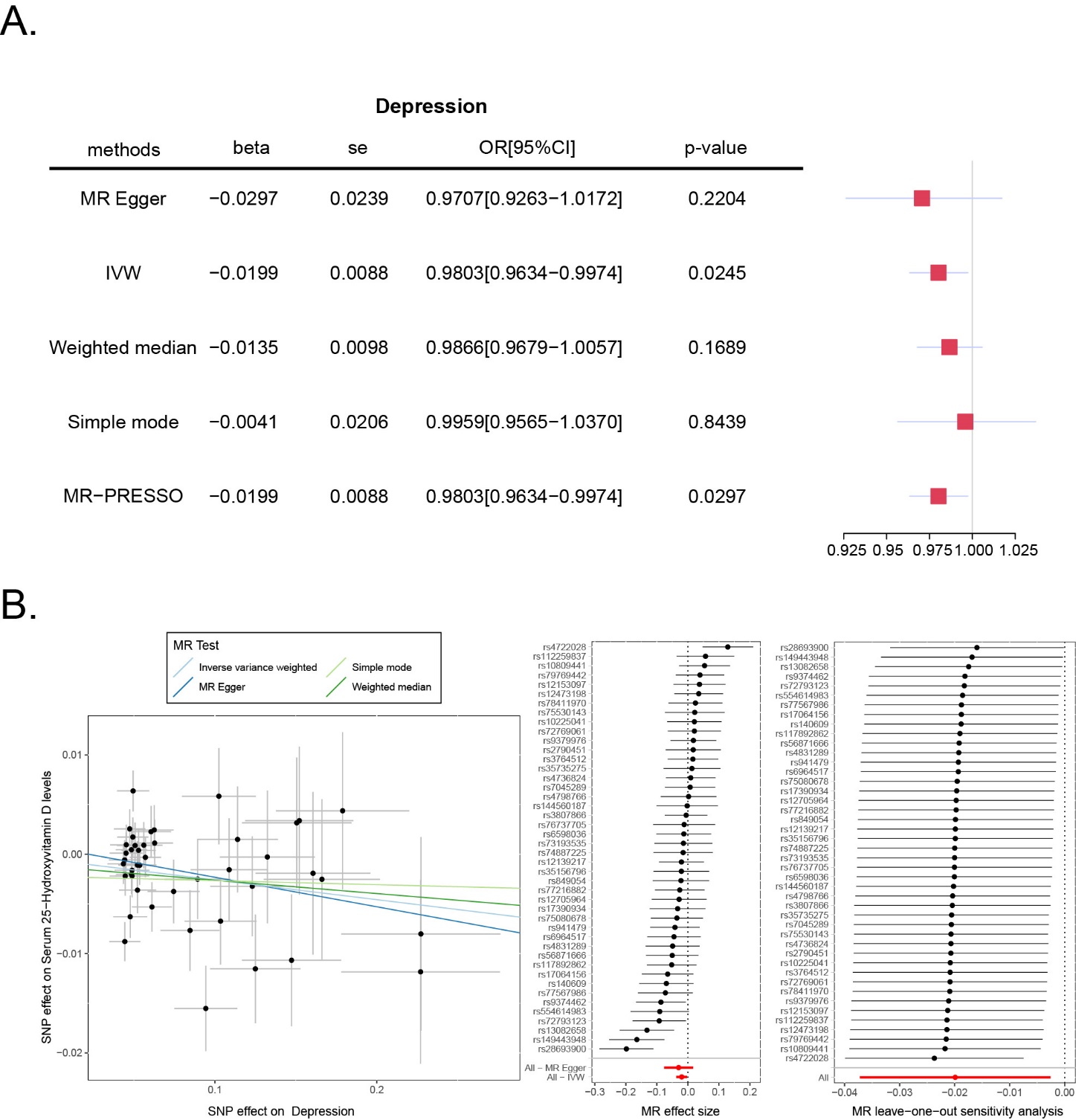


**Supplementary Figure 1. Reverse MR analysis of the effect of genetic liability to depression on serum 25-hydroxyvitamin D [25(OH)D] levels.** (A) Sensitivity analysis of causal effect estimates (OR, 95% CI) from five MR methods; (B) MR scatter plot of SNP effects and robustness checks including individual SNP forest plot and leave-one-out analysis. Note: OR = Odds Ratio; CI = Confidence Interval; se = Standard Error.


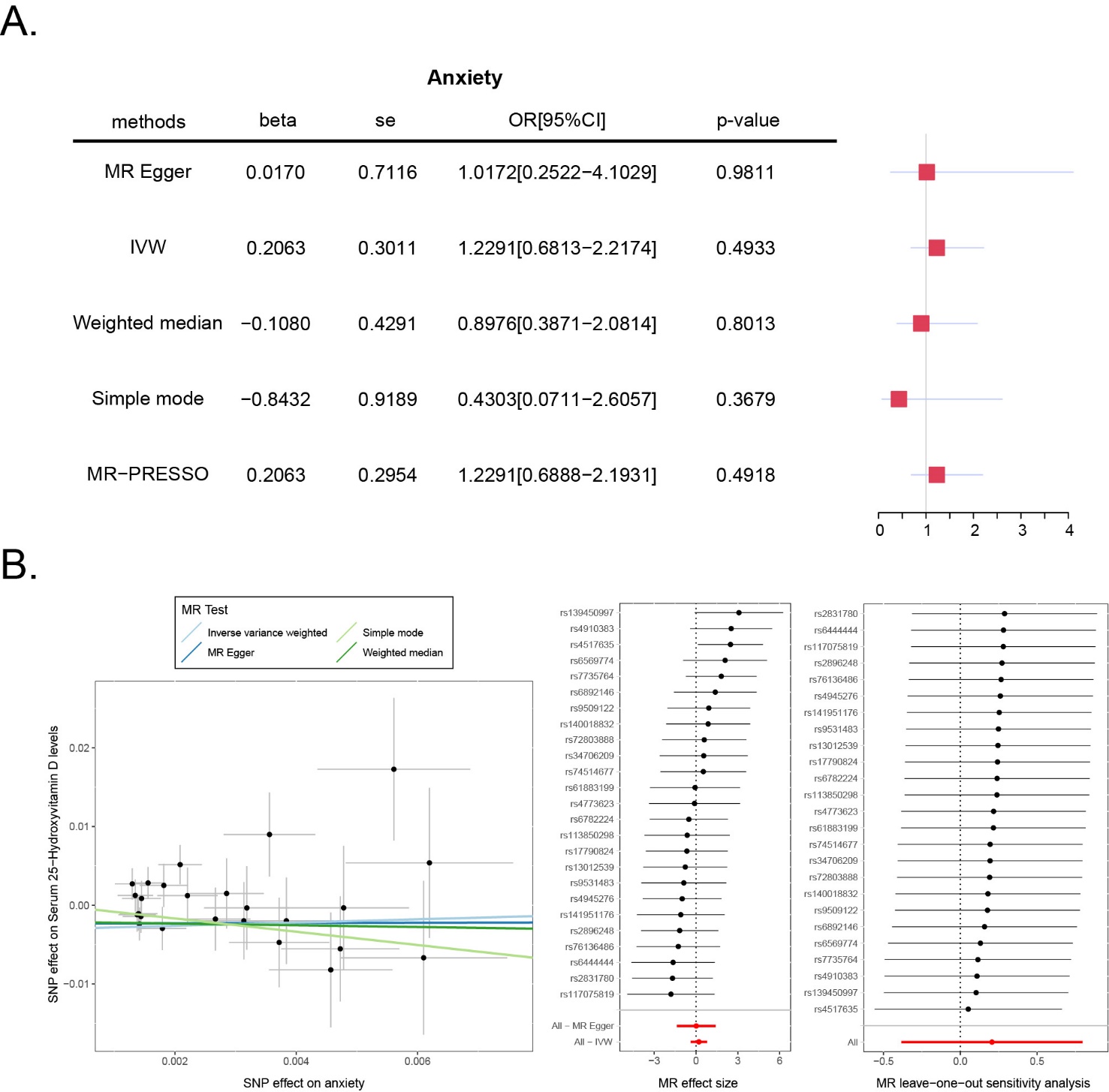


**Supplementary Figure 2. Reverse MR analysis of the effect of genetic liability to anxiety on serum 25-hydroxyvitamin D [25(OH)D] levels.** (A) Sensitivity analysis of causal effect estimates (OR, 95% CI) from five MR methods; (B) MR scatter plot of SNP effects and robustness checks including individual SNP forest plot and leave-one-out analysis. Note: OR = Odds Ratio; CI = Confidence Interval; se = Standard Error.
